# Physiopathology of Spine Metastasis

**DOI:** 10.1155/2011/107969

**Published:** 2011-08-10

**Authors:** Giulio Maccauro, Maria Silvia Spinelli, Sigismondo Mauro, Carlo Perisano, Calogero Graci, Michele Attilio Rosa

**Affiliations:** ^1^Department of Orthopaedics and Traumatology, Agostino Gemelli Hospital, Catholic University, L.go F. Vito, 1-00168 Rome, Italy; ^2^Department of Orthopaedics, Messina University, Via Consolare Valeria, 1-98122 Messina, Italy

## Abstract

The metastasis is the spread of cancer from one part of the body to another. Two-thirds of patients with cancer will develop bone metastasis. Breast, prostate and lung cancer are responsible for more than 80% of cases of metastatic bone disease. The spine is the most common site of bone metastasis. A spinal metastasis may cause pain, instability and neurological injuries. The diffusion through Batson venous system is the principal process of spinal metastasis, but the dissemination is possible also through arterial and lymphatic system or by contiguity. Once cancer cells have invaded the bone, they produce growth factors that stimulate osteoblastic or osteolytic activity resulting in bone remodeling with release of other growth factors that lead to a vicious cycle of bone destruction and growth of local tumour.

## 1. Introduction

The metastasis is the spread of cancer from one part, where it started (called its primary site) of the body to another. A tumour formed by cells that have spread is called a “metastatic tumour” or a “metastasis.” The metastatic tumour contains cells that are like those in the original (primary) tumour [1]. When cells break away from a cancerous tumour, they can travel to other areas of the body through the bloodstream or lymph system. From there, they can end up in any organ or tissue. Many of the cancer cells that break off from the original tumour die without causing any problems. Some, however, settle in a new area. There, they begin to grow and form new tumours. Sometimes metastatic tumours are found by tests that are done when the primary cancer is first diagnosed. In other cases, the metastasis is found first, causing the doctor to look for the place that the cancer started [2, 3]. 

## 2. Epidemiology

Approximately two-thirds of patients with cancer will develop bone metastasis [4]. Of the estimated 569,490 people who will die of cancer in 2010, almost all will have metastasis to some part of the body. It is estimated that about 350,000 people die with bone metastasis each year in the United States [5]. Sometimes bone metastasis is not clinically visible and their demonstration occurs during autopsy; therefore, the real incidence of bone metastasis is not possible to report [6]. Bone metastasis is actually much more common than primary bone cancers [2, 7] because the incidence is 25/1 and they are the neoplastic lesions more seen by orthopedist [8, 9]. Bones are the most common place for metastasis after lung and liver [2, 3, 10]. Primary tumors that most often leads to bone metastasis are in the order of incidence: prostate, breast, kidney, lung, and thyroid cancer [6]. The incidence of skeletal metastasis from autopsy studies is of 73% (range of 47–85%) in the breast cancer, 68% (range of 33–85%) in the prostate cancer, 42% (range of 28–60%) in the thyroid cancer, 36% (range of 30–55%) in the lung cancer, 35% (range of 33–40%) in the kidney cancer, 6% (range of of 5–7%) in the esophageal cancer, 5% (range of 3–11%) in the gastrointestinal tract cancers, 11% (range of 8–13%) in the rectal cancer [11]. Given the high prevalence of breast, prostate, and lung cancer, they are responsible for more than 80% of cases of metastatic bone disease [12]. According to Roodman GD, up to 70% of patients with breast cancer or prostate cancer, and 15 to 30% of patients with lung, colon, bladder, or kidney cancer develop bone metastasis [13]. Breast cancer is the most common malignant tumour and the main cause of bone metastasis in women [14]. About 70% of people who die from breast cancer will have radiological evidence of skeletal metastasis before their death and in 40% of cases the bone is the first metastatic site [11]; the estrogen receptors [11], the sialoprotein [15], the parathyroid-related peptide (PTHrP) [16], and 69 gene signature correlated with fibroblasts growth factors [17] are predictive markers of bone recurrence [12]. While prostate and lung metastasis are those that occur more in men [14]. The primary tumor cannot be determined in 9% of cases of spinal metastases [18].

## 3. Locations of Spine Metastasis

Metastasis can occur in any bone in the body but is most often found in bones near the center of the body. The spine is the most common site of bone metastasis [2, 12]. It is estimated that over the 10% of patients with cancer will develop a symptomatic spinal metastasis [19, 20]. Algra et al. suggest that the initial anatomic location of metastases within vertebrae is in the posterior portion of the body. Analysis of CT scans shows that the body is involved before the pedicles, although destruction of the pedicles is the most common finding on plain films. Destruction of the pedicles occurs only in combination with the involvement of the vertebral body [21]. Other common sites are the hip bone (pelvis), upper leg bone (femur), upper arm bone (humerus), ribs, and the skull [2, 14]. Studies showed that the thoracic spine is the region more involved with metastasis [22], while others studies highlighted how the lumbar spine is more involved [23, 24]. The cervical spine is the least involved (10%) [14]. More than 50% of patients with spinal metastasis have multiple levels involved, and 10 to 38% of patients have multiple, noncontiguous segments involved [14]. The lung and breast cancers metastasize preferably in the thoracic region because the venous drainage of the breast through the azygos communicates with the plexus of Batson in the thoracic region [21, 23, 25], while lung cancer drains through the pulmonary veins in the left heart and from there is distributed in the generalized manner in the skeletal; prostate cancer metastasizes usually to the lumbar-sacral spine and pelvis, because it drains through the pelvic plexus in the lumbar region [25]. Colon and rectal tumors usually metastasize through the portal system in the liver and lung, and only late in skeletal [14].

## 4. Symptoms of Bones and Spine Metastasis

Bone metastasis is one of the most frequent causes of pain in people with cancer. When a cancer spreads to the bone, it can make the bones weaker and even cause them to break without an injury [2, 7]. As the cancer cells damage the bones, calcium is released into the blood. This can lead to problems from high blood calcium levels. Bone metastasis can also cause other problems that can limit your ability to keep up your usual activities and lifestyle [2]. A spinal metastasis may cause pain, instability, neurological injuries with loss of control urinary and rectal sphincter up to paraplegia. However, 60% of all bone metastasis [26] and 36% of vertebral lesions [27] are asymptomatic and discovered occasionally. Symptomatic spinal cord involvement occurs in 18 000 patients per year [18]. Brihaye et al. analyzed 1477 cases concluded that 16.5% of spinal metastases with epidural involvement came from the breast cancer, 15.6% from the lung cancer, 9.2% from prostate cancer, and 6.5% from kidney cancer; they also analyzed 1585 cases of symptomatic epidural metastases and reported that 70.3% had involvement of thoracic and thoracolumbar region, 21.6% of the lumbar and sacral region, and 8.1% of the cervical and cervical-thoraco region, concluding that although the lumbar region is more involved, the majority of patients with neurological dysfunction have thoracic lesions [28].

## 5. Prognosis

Once cancer has spread to the bones or to other sites in the body, it is rarely able to be cured, but often it can still be treated to shrink, stop, or slow its growth. Even if cure is no longer possible, treating the cancer may be able to help you live longer and feel better [2]. The diagnosis of metastasis changes the patients' prognosis; according to data from the ACS, the survival rate at five years in nonmetastatic carcinomas treated from 1996 to 2002 was of 100% in prostate cancer, 97% in the thyroid cancer, 89% in the breast cancer, 66% in the kidney cancer, and 16% in the lung cancer; in the same period, in the metastatic tumors at presentation, the five-year survival rate was of 56% in thyroid cancer, 33% in prostate cancer, 26% in breast cancer, 10% in renal cancer, and 2% in lung cancer [29].

## 6. Method of Dissemination

**Figure 1:**
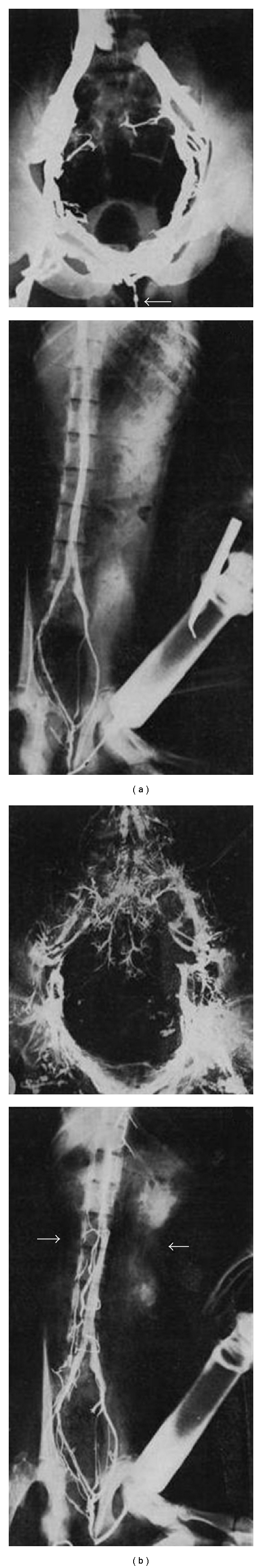
Batson venous plexus, from Batson O.V., “The function of the vertebral veins and their role in the spread of metastases,” *Ann Surg. *1940 July; 112 (1): 138–149.

The cancer can metastasize in the bone through different ways of propagation: the most frequent is the hematogenous way, the intravenous one for lesions of the spinal column, and the arterial one for lesions that at the beginning are proximal (shoulder and pelvis) and then distal (elbow and knee). Less frequent lesions are those ones by contiguity and even less frequent are those ones for lymphatic spread (whose role is not well defined) [6, 14]. The diffusion through the venous system is the principal process of spinal metastasis. In 1940, Batson (Figure 1) demonstrated by injecting contrast into the vein of the penis in males and into the veins of the breast in women that the contrast and so the tumor cells spread in the blood into the spinal veins as a result of venous reflux that occurred after an increase of intrathoracic pressure and/or intra-abdominal as for a Valsalva maneuver [30]. It was an explanation of the possibility of the diffusion of breast cancer in the column that is drained mainly by the azygos vein which communicates with the paravertebral venous plexus of Batson in the thoracic region and prostate cancer that is drained from the venous plexus which communicates with the pelvic plexus of Batson at the lumbar [31]. This hypothesis was confirmed by the study of Coman and DeLong, who noted that lumbar spinal tumor metastasis appeared in 70% of the animals, injecting cancer cells into the femoral vein of rats, when an external abdominal pressure was carried out [23, 32]. The venous plexus of Batson is a system of veins located in the epidural space between the spinal column bone and the dura mater, with no valves that control the flow of blood, so that each increase of pressure in the system of the vena cava results in an increased flow level of the plexus. It is connected to the portal and caval system that in normal conditions deviate 5–10% of blood in the vertebral venous system and with the latter [14, 23, 30, 33, 34]. Cancer cells may metastasize through the blood system and into the vertebral body directly through the nutrient arteries as in the case of lung cancer [14, 35]. Arguello et al. showed that the injection of a variety of tumor cells into the systems arterial circulation of mice resulted in a syndrome of tumor colonization of the vertebra followed by a spinal cord compression [36]. The direct diffusion of prostate cancer at the lumbar spine and the direct diffusion of the breast and lung ones at the thoracic spine are other methods of spreading [14]. 

## 7. Mechanism of Localization of Metastases in Bone

The development of a bone metastasis is not a simple process of transport, arrest, and growth of cancer cells in these spaces. Before moving to the bone marrow and taking root and growing in its spaces, neoplastic cells have to follow a long route [37]. They must first spread through the primary site at the expense of the preexisting cells and stroma then detach from it by the reduction of adhesion molecules and the opening of the epithelial basal lamina, afterwards reach the blood vessels and penetrate into them by degradation of their basal lamina and endothelium, then migrate with the bloodstream and escape the surveillance of the immune cells, reach the bone marrow sinusoids, stop and grow there [38, 39]. These processes mainly occur through the activity of proteinases, such as the metalloproteinases, the serine, cysteine, and aspartic proteinases [40–53], stromelysin [54], uPA [55, 56]. These proteinases destroy the epithelial basal lamina and the surrounding tissue by degradation of type IV collagen, laminin, proteoglycans, and other proteins but also uncover hidden biologic activities and reduce cell-to-cell adhesion by interfering with adhesion receptors in the cell membrane [47, 57]. Tumour-host interactions are mediated by a number of cell surface adhesion molecules which belong to the four superfamilies of integrins, cadherins, immunoglobulins, and selectins. The acquisition of invasive and diffusive properties by cancer cells are clearly connected with changes in these molecules, especially a fall in the expression of E-cadherin and a rise in that of CD44 [58]. The expression of adhesion molecules such as integrins *α*IIb*β*3 and *α*L*β*2, or PECAM-1, ICAM-1 and N-CAM, plays a relevant role in the interaction of cancer cells with the endothelium and matrix [59–61]. Preferential localization in skeletal segments which contain red bone marrow (vertebral bodies, ribs, iliac bones, the sternum, the femoral head, the epiphysis of long bones) can be explained by the fact that the rich vascularity allows cancer cells to be transported to this level and reduced blood flow velocity [62], together with the formation of vortices and/or microthrombi, promotes the adhesion and immobilization of the tumour cells on the endothelial ones. Another theory suggests that neoplastic cells migrate to and localize in a preferential target tissue because that is where they find the most fertile “soil” in which to grow, because the bone and bone marrow cells contain and express a variety of growth factors, cytokines, enzymes, and hormone-like substances which, together with similar factors produced by cancer cells, can make the bone microenvironment (the “soil”) suitable for cellular implantation (the “seeding”) and development [39, 63–66]. MMPs, BSP, and OPN play a key role in the implantation of neoplastic cells in bone marrow by degrading the extracellular matrix modifying cell-cell and cell-matrix contacts and interactions regulation of attachment and chemotactic migration of endothelial cells, and the promotion of angiogenesis [40, 49, 57, 67, 68]. After their localization in bone marrow spaces, their growth to clinically manifest metastases depends on a number of promoting or inhibiting conditions, primarily on interaction with surrounding bone and bone marrow cells, through the increased expression of adhesion molecules, the availability of space, degree of vascularity, and type of bone remodelling. The development of a metastasis obviously depends on the proliferation of neoplastic cells, but other processes are critical in this connection, primarily neo-angiogenesis [69].

## 8. Pathogenesis

The bone tissue undergoes a continuous process of resorption by the action of osteoclasts, and remodelling, through the action of osteoblasts. In normal individuals, this process is balanced. In cancer cells, this balance is lost and lytic, thickener, or mixed lesions are created [12, 13]. The osteolytic lesions are caused by stimulation of osteoclastic activity accompanied by reduced osteoblastic activity not by direct effects of tumour cells on the bone [70, 71]. The osteoblastic lesions are expression of an increased bone formation around the tumour cells associated with a disequilibrium of the osteolytic activity and with an altered turnover of the bone [71]. Once cancer cells have invaded the bone, they produce growth factors that directly stimulate osteoclastic activity and/or osteoblastic activity resulting in bone remodelling and further release of growth factors that lead to a vicious cycle of bone destruction and growth of local tumour [13, 71, 72].

## 9. Osteolytic Metastasis Pathogenesis

**Figure 2:**
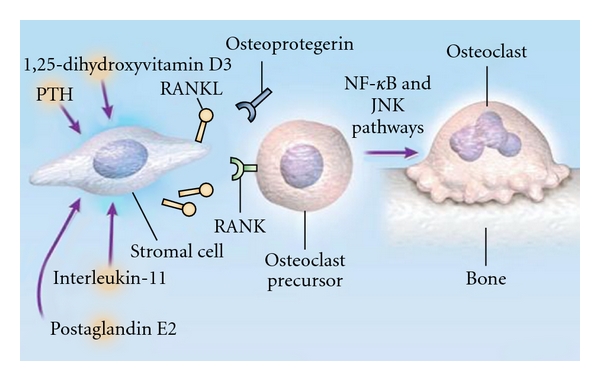
Receptor Activator of Nuclear Factor *k *B Ligand (RANK) and Osteoclast Formation, from Roodman G. D., “Mechanisms of bone metastasis,” *N Engl J Med.*, 15; 350 (16): 1655–64, Apr 2004.

**Figure 3:**
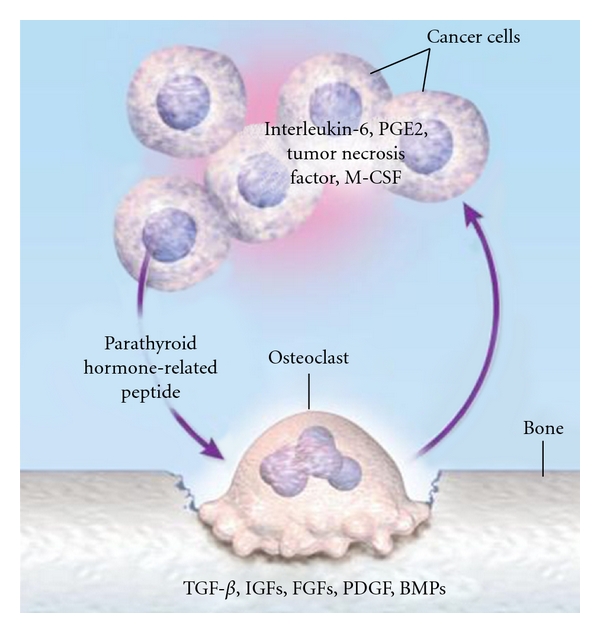
The Vicious Circle of Osteolytic Metastasis, from Roodman G. D., “Mechanisms of bone metastasis,” *N Engl J Med.*, 15; 350 (16): 1655–64, Apr 2004.

Tumour cells produce IL-1-6-8-11, PgE2, TGF*α*, TGF*β*, EGF, VEGF, TNF, CSF-1, GM-CSF, and M-CSF, which can directly or indirectly stimulate osteoclastic activity and then bone resorption [5, 12, 13, 72, 73]. Proteolytic enzymes, as acid phosphatase, acid hydrolase, alkaline phosphatise [74], metalloproteinase MMP-2, MMP-9, and K cathepsin seemed to be involved in the early phase of bone metastasis formation degrading bone basal membrane, facilitating tumoral diffusion and bone matrix cytokine release and stimulating tumour cell proliferation [75]. Tumour cells may increase bone resorption also stimulating the tumour-linked immune response with release of osteoclastic activating factors [76]. PTHrP produced by breast cancer cells plays a key role in bone resorption stimulating osteoclastic activity [77, 78]; it is more present in metastatic breast cancer (92%) than in not metastatic ones (50% ) [79]. PTHrP and IL 1-6-11 induce osteoclastic bone resorption stimulating osteoblasts and stromal cells to produce the receptor activator of nuclear factor-kB (RANK) ligand; this factor links to its receptor on the osteoclastic precursors inducing their proliferation and differentiation (Figure 2) [76]. The bone damage consequently obtained facilitates the growth factors release causing tumour cells proliferation, as TGF*β*, IGFs, FGFs, PDGF, BMPs, which stimulates PTHrP production and then osteolysis [12, 80]. So a vicious circle is present (Figure 3): osteolysis and growth factors release stimulate tumour cells proliferation and then metastatic cells growth [72, 80]. Usually OPG production by osteoblasts neutralizes RANK ligand locking osteclastic stimulation, but it has been demonstrated that OPG release is reduced in MCF-7 estrogen-dependent breast cancer cell line stimulating also osteoclastic activity [81]. Also IL-6 expressed in prostate and breast cancer cells stimulates osteoclasts cells strengthening the effects of PTHrP onto osteoclasts [82, 83].

## 10. Osteoblastic Metastasis Pathogenesis

Bone blastic metastasis is usually present in prostate cancer. Growth factors as TFG*β*, PDGF, BMPs, IGFs, FGFs, and l'u-PA (which stimulates TGF*β* release) have been isolated in prostate cancer cells and stimulate osteoblastic differentiation and they have a role in growing and survival tumour cells itself [70, 74, 84, 85]. It has been demonstrated that endothelin 1 level is elevated in bone metastatic prostate tumours than in nonmetastatic ones [86]. It stimulates osteoblastic activity and inhibits the osteoclastic one [87], increases cancer cells proliferation, and stimulates the other growth factors mitogen effects [88]; its production is reduced by androgens and is increased in the androgen-resistant diseases [89]; it is important because usually prostate cancer develops androgene resistance. ET-1 antagonists reduce either osteoblastic bone metastatic growth or tumour growth [90]. Also PTHrP and its receptor have been found in bone metastases and in primary prostate cancer, and it has been demonstrated that prostate tumour cells are able to directly express a form of RANK ligand, which directly induces bone resorption [91], revealing that osteolytic activity is present in prostate cancer [92]. Bone degradation products have been found in urine leading to the hypothesis that in prostate cancer there is at the beginning an osteolytic activity followed by high osteoblastic one [93]. Another study demonstrated that the insertion of PC-3 tumour cells in SCID mice tibia caused osteolytic lesions due to RANK ligand, while other cell lines caused osteoblastic ones, so authors reported that osteoclastic activity is not a prerequisite for osteoblastic lesions [94]. Further study is necessary for this [13]. Moreover, in prostate cancer Wnt induces osteoblastic activity, that in the early phase may be balanced by DKK1 Wnt agonist (an osteoblastic differentiate inhibitor), leading to lythic lesions. After the tumour progression, the balance between Wnt and its inhibitors is shifted towards the first, promoting osteoblastic lesions [95, 96]. Nevertheless, PSA tumour-induced can block PTHrP [97] and then bone resorption and activating osteoblastic growth factors as TGF*β*, l'IGF-1 released by bone during metastastic development, leading to a vicious circle also for osteoblastic lesions [13]. 

## Abbreviations

ACS: American Cancer Society
PTHrP: Parathyroid-related peptide
uPA: Urokinase-tipe plasminogen activator
MMPs: Matrix metalloproteinases
BSP: Bone sialoprotein
OPN: Osteopontin
IL: Interleukins
PGE2: Prostaglandin E2
TGF: Transforming growth factor
EGF: Epidermal growth factor
VEGF: Vascular endothelial growth factor
TNF: Tumor necrosis factor
CSF: Colony stimulating factor
GM-CSF: Granulocyte macrophage-colony stimulating factor
M-CSF: Monocyte-colony stimulating factor
RANK: receptor activator of nuclear factor
IGF: Insulin-like growth factor
FGF: Fibroblast growth factor
PDGF: Platelet-derived growth factor
BMP: Bone morphogenetic protein
OPG: Osteoprotegerin
ET: Endothelin.
